# Supplementary material for: A Glutathione Peroxidase Gene from Litopenaeus vannamei Is Involved in Oxidative Stress Responses and Pathogen Infection Resistance
Source: Int J Mol Sci. 2022 Jan 5;23(1):567. doi: 10.3390/ijms23010567 (PMC8745291; doi:10.3390/ijms23010567)
Supplement: Supplementary file 1 [file ijms-23-00567-s001.zip › ijms-1530891-supplementary.pdf]

```
1     cgccggttataaggccggggccgggacaggagagacaagacttgtgagcttcagtttgcg
61    tccagctactagacgggaaggtagcggtcgtctctctgctcgcagcATGTTGTGGGCGGG
1                                                      M  L  W  A  G
121   GTTCGTGTCCCTCGCGGCCCTGAGTGTGGGCGCGGGGGCGGCGTCGGATCGGGTCGCGGC
6      F  V  S  L  A  A  L  S  V  G  A  G  A  A  S  D  R  V  A  A
181   GAGGGCGTGCTTCCACCACCCCAACAACGGCGGCACCATCTATGACTTCAACGAGTTGGA
26     R  A  C  F  H  H  P  N  N  G  G  T  I  Y  D  F  N  E  L  D
241   CCTCTTCGAGACGAGGAACGTTTCCCTCGCCGACTACAAGGGGAAGGTAGTGTTGATCGT
46     L  F  E  T  R  N  V  S  L  A  D  Y  K  G  K  V  V  L  I  V
301   CAATGTGGCGACCTACTGNGGGTACACTGCTCAGTACCTCGACATGAATGTGCTACAAAA
66     N  V  A  T  Y  X  G  Y  T  A  Q  Y  L  D  M  N  V  L  Q  N
361   CTCTTACAACAACTTTGAGATCCTGGGCTTCCCTTGCAACCAATTCGGGAAGCAAGAGCC
86     S  Y  N  N  F  E  I  L  G  F  P  C  N  Q  F  G  K  Q  E  P
421   GGCAGCGAACGCCACGGAGCTCTATAACGGGATCAAGTACGTTCGCCCGGGCAACATGTT
106    A  A  N  A  T  E  L  Y  N  G  I  K  Y  V  R  P  G  N  M  F
481   CGAGCCAAATTTCACTCTGTTCAAGAAGATCGAAGTGAACGGCGAGAATGAACACCCGCT
126    E  P  N  F  T  L  F  K  K  I  E  V  N  G  E  N  E  H  P  L
541   GTACACTTACCTCAAGGAGTACTGCCCGACCACCAGGGAGTCCTTTTCGGATAAGTCGAA
146    Y  T  Y  L  K  E  Y  C  P  T  T  R  E  S  F  S  D  K  S  K
601   GCTGTACTACGAGCCCGTCCGCATCAGTGACGTCCGCTGGAACTGGGAGAAGTTCCTGAT
166    L  Y  Y  E  P  V  R  I  S  D  V  R  W  N  W  E  K  F  L  I
661   CACCAAGAGCGGCAAGCCGTTCATGCGGTACGACCCTGGCACCAAGCCGGAAGAGATCAA
186    T  K  S  G  K  P  F  M  R  Y  D  P  G  T  K  P  E  E  I  K
721   GAACGACGTGTTGTTCCTCCTGAGCCAGGAATTTTAAgtttttttttcttgtttttattt
206    N  D  V  L  F  L  L  S  Q  E  F  *
781   tttattattatttttttttttttcatctgaaggcttgatctttttgttaattttgatgtaa
841   gtgtgtgaactttttttgataacttgacagtcatgttgctactgctgcttcgtgtgtctct
901   tgtgccttccttgtgaccgctgggagtccgcgccttcgcccatgcatggtgccatgaagg
961   tgccgccctaaaccccccgggtgggcggcgctggaaggcgggaggagctcgggtcgggtc
1021  ttctagcacattcatcctgacccctgtgacctctgccggccatgcgggggggtcaaaggtc
1081  acggcgacgactcaaaacactagagtaaatgcacttttactttcgagttgcttttggtgc
1141  atgaatttctttttgtcttttttttttttttttttttttttgtctttttttctattttttttt
1201  ttcttttttttatccgaaggcttgatctttttgttaattttgatgtaagtgtgtgaacttt
1261  tttttgataacttgacagtcatgttgctactgctgcttcgtgtgtctcttgtgccttcct
1321  tgtgaccgctgggagtccgcgccttcgcccatgcatggtgccatgaaggtgccgccctaa
1381  accccccgggtgggcggcgctggaaggcggggggagctcgggtcgggtcttctagcacat
1441  tcatcctgacccctgtgacctctgccggccatgcgggggggtcaaaggtcacggcgacgac
1501  tcaaaacactagagtaaatgcacttttactttcgagttgcttttggtgcatgaatttctt
1561  tttgtcttttttttttttttttttttttttgtctttttttctatttttttttttcttttttta
1621  tccgaaggcttgatctttttgttaat
```

Figure S1. Nucleotide and deduced amino acid sequences of LvGPX3. The ORF of the nucleotide sequences are shown in upper-case letters, the 5′and 3′-UTRs are shown in lower-case letters. Nucleotides and amino acids are numbered on the left of the sequences. The initiation codons are in bold. The signal peptide are in red. And the GSHPx domain are shaded in gray.

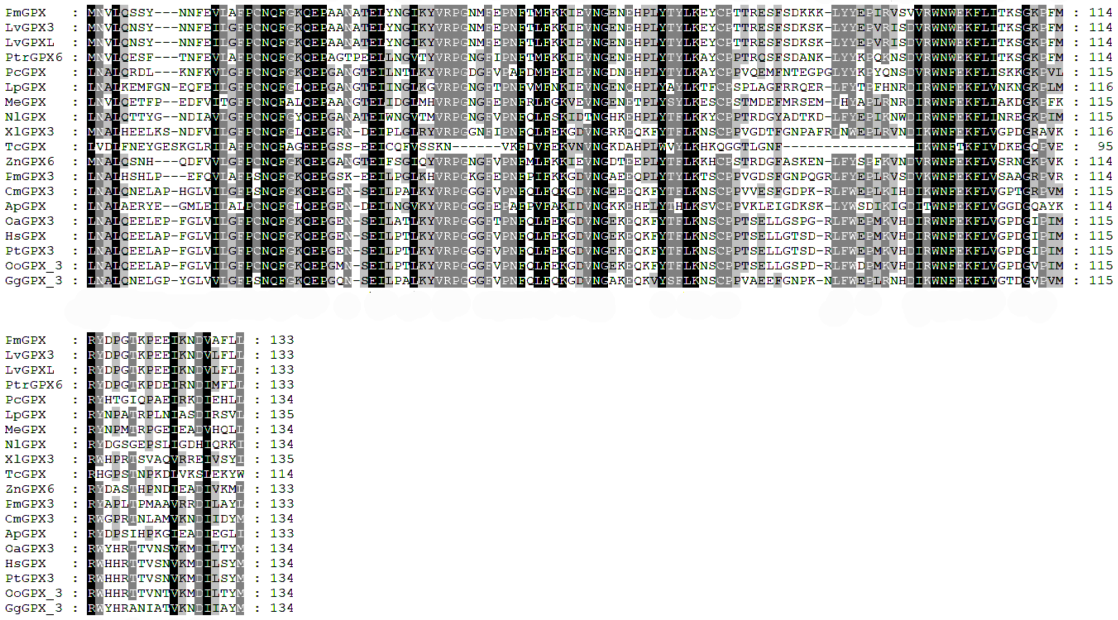

Figure S2. Multiple-sequence alignment of the deduced amino acid sequence of GPXs. The sequences were aligned by Clustal X 2.0. Completely conserved residues across all species aligned were shaded in black, and residues with less conserved were shaded in gray.

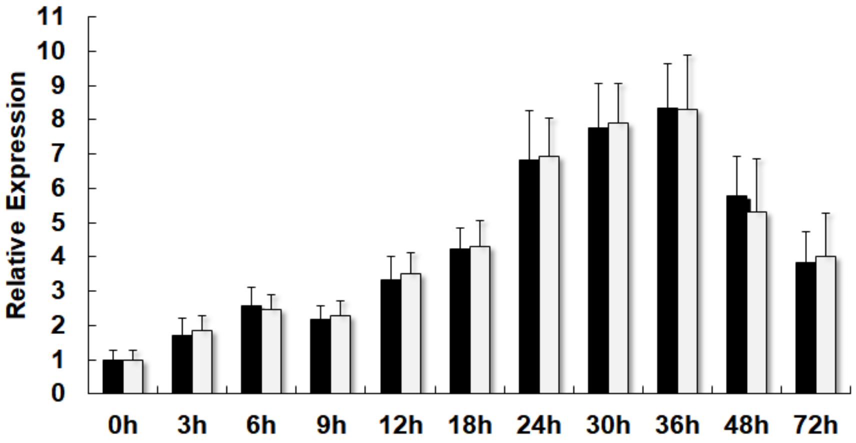

Figure S3. Influence LvGPX3 on *Crustin* expression under LPS treatment. Upon LPS treatment, knockdown expression of *LvGPX3* did not significant influenced the expression of *Crustin* (Genbank accession no. MG883729). For LPS injection, each shrimp was injected with LPS 240 μg that dissolved in 50 μL PBS 36 h after dsRNA injection. The bars represent the mean values ± S.D. of three replicates.
